# p53 isoform Δ133p53 promotes efficiency of induced pluripotent stem cells and ensures genomic integrity during reprogramming

**DOI:** 10.1038/srep37281

**Published:** 2016-11-22

**Authors:** Lu Gong, Xiao Pan, Haide Chen, Lingjun Rao, Yelin Zeng, Honghui Hang, Jinrong Peng, Lei Xiao, Jun Chen

**Affiliations:** 1 Key laboratory for Molecular Animal Nutrition, Ministry of Education, Innovation Center for Signaling Network, College of Life Sciences, Zhejiang University, Hangzhou 310058, China; 2 Department of Genetics and Complex Diseases, Harvard School of Public Health, Boston, Massachusetts 02115, USA; 3 College of Animal Sciences, Zhejiang University, Hangzhou 310058, China; 4 Key Laboratory of Freshwater Fish Reproduction and Development, Ministry of Education, State Key Laboratory Breeding Base of Eco-Environments and Bio-Resources of the Three Gorges Reservoir Region, School of Life Sciences, Southwest University, 2 Tiansheng Road, Beibei, Chongqing, 400715 China.

## Abstract

Human induced pluripotent stem (iPS) cells have great potential in regenerative medicine, but this depends on the integrity of their genomes. iPS cells have been found to contain a large number of *de novo* genetic alterations due to DNA damage response during reprogramming. Thus, to maintain the genetic stability of iPS cells is an important goal in iPS cell technology. DNA damage response can trigger tumor suppressor p53 activation, which ensures genome integrity of reprogramming cells by inducing apoptosis and senescence. p53 isoform Δ133p53 is a p53 target gene and functions to not only antagonize p53 mediated apoptosis, but also promote DNA double-strand break (DSB) repair. Here we report that Δ133p53 is induced in reprogramming. Knockdown of Δ133p53 results 2-fold decrease in reprogramming efficiency, 4-fold increase in chromosomal aberrations, whereas overexpression of Δ133p53 with 4 Yamanaka factors showes 4-fold increase in reprogamming efficiency and 2-fold decrease in chromosomal aberrations, compared to those in iPS cells induced only with 4 Yamanaka factors. Overexpression of Δ133p53 can inhibit cell apoptosis and promote DNA DSB repair foci formation during reprogramming. Our finding demonstrates that the overexpression of Δ133p53 not only enhances reprogramming efficiency, but also results better genetic quality in iPS cells.

Human induced Pluripotent Stem (iPS) cells can be generated by viral-based ectopic expression of specific transcription factors (e.g., Oct4, Sox2, Klf4, and c-Myc), which provides great potential for use in research and regenerative medicine. However, a number of studies have shown that the reprogramming process can induce genetic abnormalities in iPS cells^1^,^2^,^3^,^4^,^5^,^6^. More than 1000 heterozygous single-nucleotide variants were found in human iPS cell lines induced even by non-integrating plasmid expression^3^. These studies raise great concerns on the chromosome aberrations for future application of iPS cells. The most possible reason for generation of genetic variants in iPS cells is that early reprogramming of iPS cells induced by Yamanaka factors triggers the DNA damage response^7^,^8^. A method for maintaining the genetic stability of iPS cells is very crucial for practical application.

Tumor suppressor p53 is activated by DNA damage and plays a central role in the DNA damage response. The activation of p53 induces cell cycle arrest, DNA damage repair, apoptosis and/or senescence to guard genome stability. Previous studies showed that the p53 signal pathway is activated and DNA double-strand break (DSB) repair foci are formed during cell reprogramming, which suggests that the process of cell reprogramming causes DNA DSBs^7^,^9^,^10^. The most toxic lesion in DNA is the DSB. To combat this toxic insult, a number of pathways have evolved to repair DNA DSBs: Homologous Recombination (HR), Non-Homologous End Joining (NHEJ) and Single-Strand Annealing (SSA). In contradict to its tumor suppression role, p53 protein inhibits the HR, NHEJ and SSA pathways^11^. It has demonstrated that p53 plays a dual role in iPS cell reprogramming^12^. Early reprogramming of iPS cells induced by Yamanaka factors triggers the DNA damage response which activates p53. The activated p53 prevents the reprogramming of cells carrying various types of DNA damage by promoting apoptosis and senescence of these cells^7^,^9^,^10^. Although the knockdown of p53 allows high reprogramming efficiency, the generated iPS cells have a high risk of carrying DNA aberrations^7^,^13^.

∆133p53, an N-terminal truncated isoform of p53, is transcribed by an alternative *p53* promoter in intron 4^14^,^15^. Previous studies showed that ∆133p53 is a p53 target gene and functions to antagonize p53 apoptotic activity by differentially modulating expression of p53 target genes^16^,^17^,^18^. The anti-apoptotic activity of Δ113p53 (Δ133p53 ortholog in zebrafish) is dependent on the protein interaction between p53 and Δ113p53^19^. On the otherhand, ∆133p53 can also coordinate with p53 to promote cell survival under sub-toxic oxidative stresses via promoting anti-oxidant gene expression^20^. In a recent study, we demonstrate that Δ133p53 is strongly induced by DNA DSBs and not only inhibits cell apoptotic activity, but also promotes all three DNA DSB repair pathways to protect cells from death and DNA damages upon γ-irradiation. Δ133p53 promotes DNA DSB repair via up-regulating the transcription of the three DNA DSB repair genes: *RAD51*, *LIG4* and *RAD52*, independent of p53^21^.

Here, we demonstrate that Δ133p53 is induced during cell reprogramming to promote reprogramming effciency and ensure genomic integrity of iPS cells.

## Results

### Δ133p53 is induced in cell reprogramming and functions to promote reprogramming efficiency

**Figure 1:**
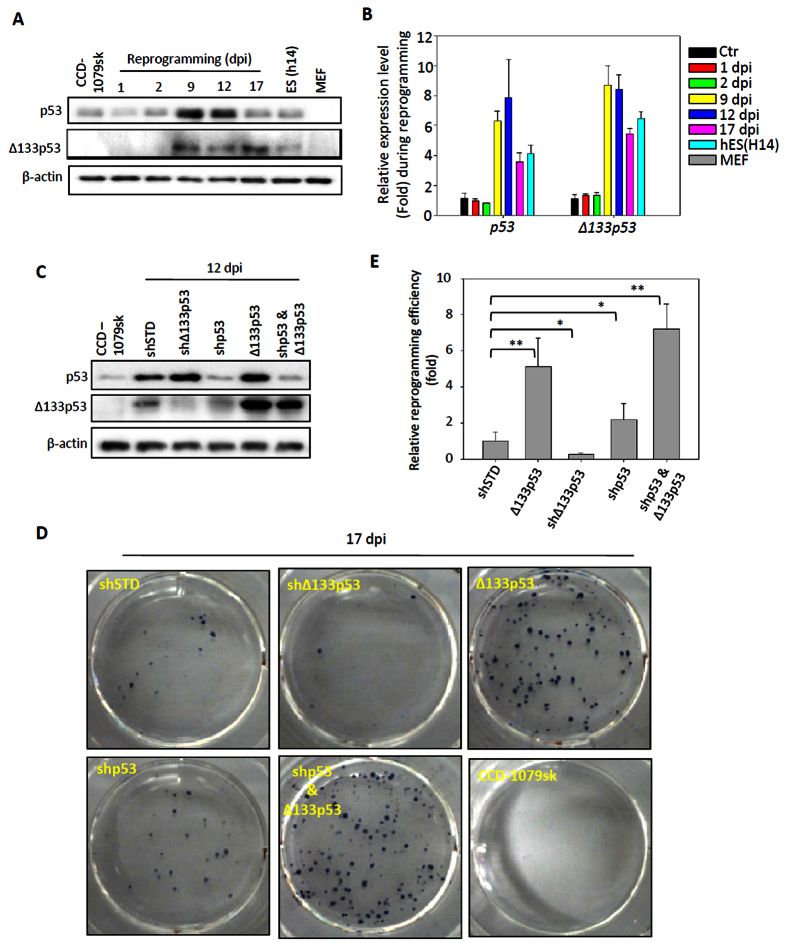
Induction of p53 and Δ133p53 in human induced pluripotent stem (iPS) cell reprogramming. (**A**) Western blot of p53 and Δ133p53 from human fibroblast CCD-1079sk cells reprogrammed with 4 Yamanaka factors at different day post infection (dpi). Human embryonic stem cells (line h14) [ES (h14)] were used as a stem cell control; Feeder cells (mouse embryonic fibroblast: MEF) was used as a negative control. β-actin was loading control. (**B**) qRT-PCR to analyze p53 and Δ133p53 transcripts from reprogrammed human fibroblast CCD-1079sk cells at different dpi. Total RNA was sampled from human fibroblast CCD-1079sk cells reprogrammed with 4 Yamanaka factors at dpi as indicated and subjected to qRT-PCR. Expression levels of analyzed genes were normalized against β-actin. (**C**) Western blot was performed to show knockdown of p53 and Δ133p53 with specific shRNA and over-expression of Δ133p53 in reprogramming cells at 12 dpi. (**D**) Reprogramming plates stained with alkaline phosphatase (AP) at 17 dpi. CCD-1079sk cells were infected by four Yamanaka factors in combination with different constructs as indicated. (**E**) Statistical analysis from three repeat experiments was shown in (**C**).

The role of Δ133p53 in DNA DSB repair prompted us to speculate that Δ133p53 may have an effect in cell reprogramming. We first checked the expression of Δ133p53 at 1, 2, 9, 12 and 17 days post infection (dpi) during the reprogramming of human skin fibroblast (CDD-1079sk) cells mediated by the four Yamanaka factors. Interestingly, we found that Δ133p53 protein and transcript were induced, as were those of full-length p53, from 9 dpi (Fig. 1A,B). Notably, Δ133p53 was also expressed in the human embryonic stem cells (embryonic cell line 14)^22^, but not in mouse embryonic fibroblast (MEF) cells (Fig. 1A,B). Next, we combined the four Yamanaka factors with specific short hairpin RNAs (shRNA) to knockdown p53 or Δ133p53, or used *ef1a-Δ133p53* to overexpress Δ133p53 during reprogramming (Fig. 1C). The knockdown and ectopic expression of Δ133p53 did not have much effect on the level of full-length p53 protein (Fig. 1C). However, the knockdown of full-length p53 also downregulated the expression of Δ133p53 (Fig. 1C), which is consistent with that Δ133p53 is a p53 target gene. Similar to the previous studies^7^, the knockdown of full-length p53 promoted the reprogramming efficiency in an approximately 2-fold increase in compared to the control cells co-infected with a nonspecific shRNA (shSTD) (Fig. 1D,E). In contrast, the knockdown of Δ133p53 resulted in a 2-fold decrease and the overexpression of Δ133p53 showed a 4-fold increase in reprogramming efficiency (Fig. 1D,E). Combining the knockdown of p53 and the overexpression of Δ133p53 resulted in a further 4-fold increase compared to the knockdown of p53 alone (Fig. 1D,E). These results demonstrate that Δ133p53 promotes iPS cell reprogramming.

### Overexpression of Δ133p53 inhibits apoptosis during reprogramming

**Figure 2:**
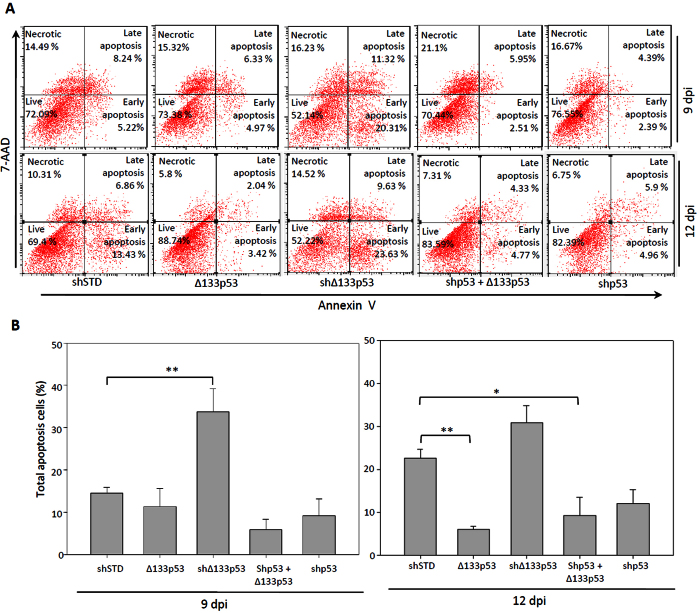
Δ133p53 inhibits apoptosis in cell reprogramming. (**A**) Representative FACS profiles at 9 and 12 dpi. CCD-1079sk cells were infected by four factors in combination with different constructs as indicated. Reprogramming cells were stained with 7-aminoactinomycin (7-AAD) and Annexin V and subjected to FACS analysis. (**B**) Statistic analysis of total apoptotic cells in different samples (including early and late apoptotic cells) as shown from three repeat experiments.

To investigate whether the increase of iPS cell reprogramming efficiency is correlated with Δ133p53’s anti-apoptotic activity, we performed a fluorescence-activated cell sorting (FACS) analysis with anti-Annexin V antibody staining at 9 and 12 dpi. The results showed that the percentage of reprogramming cells undergoing apoptosis at 9 dpi was significantly increased more than 2 folds in the treatment with the knockdown of Δ133p53, whereas the percentage of apoptotic cells was slightly decreased in the treatments with either the overexpression of Δ133p53 or the knockdown of p53, compared to that in the control reprogramming cells co-infected with shSTD (Fig. 2A,B). The analysis from 12 dpi showed that the percentage of apoptotic cells was 5.46% four-fold lower in the treatment with the overexpression of Δ133p53 and was 10.86% two-fold lower in the treatment with the knockdown of p53, whereas the percentage of apoptotic cells was increased about 8% in the treatment with the knockdown of Δ133p53, compared to that (20.23%) in the control treatment (Fig. 2A,B). The percentage of apoptotic cells (9.01%) in the treatment with combining the knockdown of p53 and the overexpression of Δ133p5*3* was higher than that in the treatment with the overexpression of Δ133p53 alone, but still two-fold lower than that in the shSTD co-infected control group. Nevertheless, the results suggest that one of reasons for Δ133p53 to promote reprogramming efficiency is inhibition of apoptosis.

### Δ133p53 promotes DNA DSB repair in cell reprogramming

**Figure 3:**
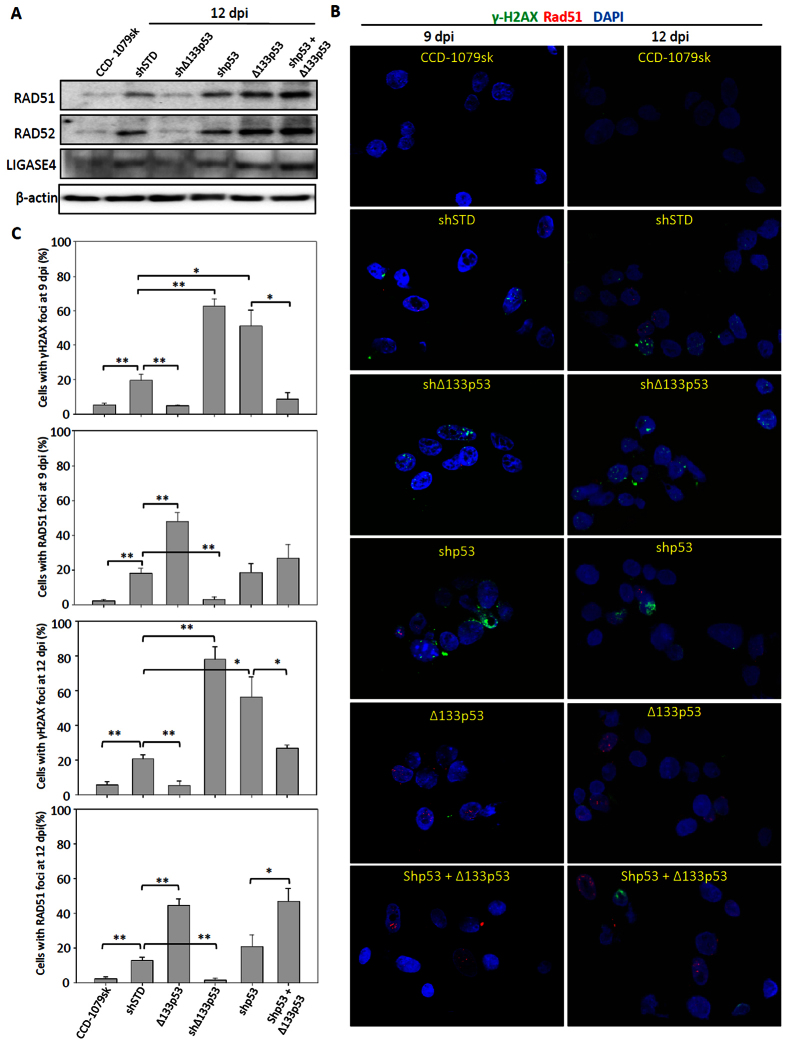
Δ133p53 promotes DNA DSB repair during reprogramming. (**A**) Western blot analysis of RAD51, LIG4 and RAD52 in reprogramming cells. Total proteins were extracted from CCD-1079sk reprogramming cells with different treatments at 12 dpi as indicated and subjected to Western blot analysis with specific antibodies respectively. (**B**) Co-immunostaining of RAD51 (in red) and γH2AX (in green) in CCD-1079sk reprogramming cells with different treatments at 9 and 12 dpi as indicated. DAPI was used to stain the nuclear DNA (blue). (**C**) Statistical analysis of the average number of RAD51 or γH2AX positive cells (including cells with foci formation and pan nuclear signal) in different samples as shown in E. About 150 cells in each sample were randomly picked up for counting RAD51 or γH2AX positive cells. Statistical analysis was performed based on the data from three repeat experiments.

Previous reports showed that DNA DSB repair foci are formed during cell reprogramming^7^,^8^, which suggests cell reprogramming can induce DNA DSBs. Our recent finding demonstrated that Δ133p53 promotes DNA DSB repair by upregulating the expression of RAD51, LIG4 and RAD52^21^. Therefore, we checked the protein accumulation of these three genes in reprogramming at 12 dpi using Western blot (Fig. 3A). The results showed that three DNA DSB repair genes, RAD51, LIG4 and RAD52 were all up-regulated at 12 dpi after reprogramming (Fig. 3A). The expression of these genes after reprogramming was down-regulated by the knockdown of Δ133p53 and enhanced by overexpression of Δ133p53 (Fig. 3A). The results suggested that cell reprogramming triggers DNA DSB response and Δ133p53 may promote DNA DSB repair during cell reprogramming.

Next, we investigated the function of Δ133p53 in the formation of the DNA DSB repair foci of phosphorylated H2AX (γH2AX) and RAD51 at 9 and 12 dpi during reprogramming. γH2AX is one of the early DNA DSB repair markers. RAD51 is a recombinase and required for HR repairs which executes high fidelity DNA repair by using the undamaged sister chromatid or homologous DNA as a template to faithfully repair the damage. Similar effects of Δ133p53 on DNA damage repair were observed at both 9 and 12 dpi. The proportion of cells with RAD51 positive staining (including foci and pan-nuclear signals) increased approximately 2 to 3-fold with the overexpression of *Δ133p53* and decreased almost 5 to 7-fold with the knockdown of *Δ133p53*, whereas the percentage of RAD51 positive cells was not significantly changed by the knockdown of p53, compared to that in the control cells co-infected with shSTD (Fig. 3B,C). However, the proportion of cells with γH2AX positive staining (including foci and pan-nuclear signals) was significantly increased by the knockdown of either p53 (about 2-fold) or Δ133p53 (about 3-fold), but significantly decreased by overexpression of Δ133p53 (about 3-fold), compared to the control (Fig. 3B,C). From these results, we speculated that Δ133p53 protects iPS cell genomic stability by promoting DNA DSB repair.

### Δ133p53 reduces chromosomal aberrations in iPS cells

**Figure 4:**
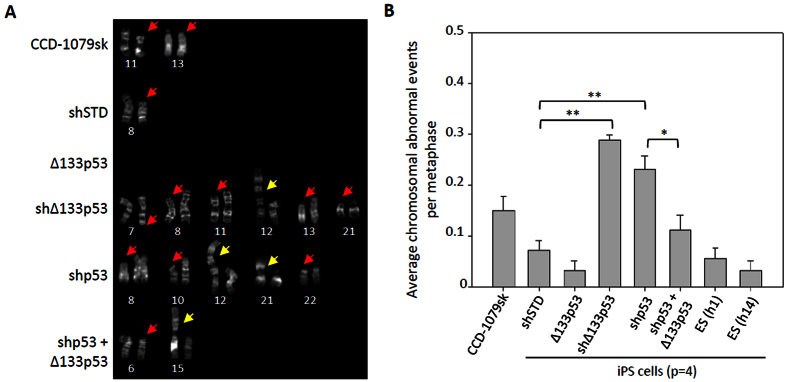
Δ133p53 reduces chromosomal abnormalities in iPS cells. (**A**) Karyotype analysis of iPS cells at passage 4. Five independent iPS clones from each treament were selected for further expansion at 25–30 dpi. In each iPS clone, 25 metaphases and about 1000 chromosomes were observed. Total chromosomal aberration events including breakage (red arrow) and end-to-end fusion (yellow arrow) were from one of five iPS clones in each treatment as indicated. (**B**) Average abnormal chromosome events came from 5 independent iPS clones in each treatment. Student’s t-test was used for statistics.

To confirm this speculation, we selected five independent iPS cell clones from each treatment and performed a chromosomal damage analysis at passage four using a karyotype assay. The characteristics of the selected iPS cell clones were confirmed by different iPS markers (Fig. S1A,B). Pluripotency of iPS cell clones from both of the control group and the treatment with co-expression of Δ133p53 was identified by the analysis of teratoma formation (Fig. S2). Chromosomal aberration events, including chromosome breakages and end-to-end fusions, indeed increased 2-fold in the iPS cells with a p53 knockdown and 3-fold in cells with a Δ133p53 knockdown, compared to that in shSTD infected controls (Fig. 4A,B). Strikingly, there were only half as many aberration events in the iPS cells overexpressing Δ133p53 as in the shSTD infected controls, even though the reprogramming efficiency in the iPS cells overexpressing Δ133p53 was increased 4-fold. The overexpression of Δ133p53 significantly decreased the chromosomal aberration events caused by the knockdown of *p53* in the iPS cells (Fig. 4A,B), which is consistent with that Δ133p53 promotes DNA DSB repair independent of p53. These data demonstrate that the genetic quality of iPS cells can be improved by the overexpression of Δ133p53.

## Discussion

*De novo* genetic variants in iPS cells have been observed in many studies^1^,^2^,^3^,^4^,^5^,^6^. To minimize the genomic instabilities of iPS cells, strategies of generating integration-free iPS cells have been developed. However, iPS cells generated either with episomal vector or protein-base method were still found to carry a large number of *de novo* genetic variants^3^,^23^. One of the most important reasons for the *de novo* genetic variants in iPS cells is that reprogramming process can trigger DNA damage response. Therefore, faithful repairing DNA damages during reprogramming is very crucial for maintenace of genomic integrity.

Tumour repressor p53, often known as the “guardian of the genome”, is a key regulator in DNA damage response. It has demonstrated that p53 inhibits cell reprogramming by promoting reprogramming cells to undergo apoptosis and senescense^7^,^9^,^10^. When p53 is absent, reprogramming efficiency is significantly increased. However, the genetic quality of generated iPS cells is getting worse^7^,^13^.

In the last decade, p53 has been found to encode a large number of isoforms^24^,^25^. It has demonstrated that p53 isoforms can modulate p53 functions either synergistically or antagonistically^26^. Our recent studies showed that Δ133p53, an N-terminal truncated p53 isoform, not only antagonizes p53-mediated apoptosis, but also promotes DNA DSB repair^21^. Here, we report that Δ133p53 is induced during reprogramming. Δ133p53 not only promotes efficiency of cell reprogramming by its anti-apoptotic function, but also ensures genetic stability by promoting DNA DSB repair. Our results imply that overexpression of Δ133p53 during reprogramming may provide a solution for improving iPS genetic quality due to its ability to increase RAD51 foci formation and decrease γH2AX foci formation and chromosome aberrations in iPS cells.

## Materials and Methods

### Construction of expression plasmids

Four lentivirus plasmids: LV-OCT4-Egfp, LV-SOX2-Egfp, LV-KLF4-Egfp, LV-CMYC-Egfp, and 2 helper plasmids: pdR8.91 and pVSVG were constructed as previously described^27^. To construct LV-Δ133p53-EGFP, Δ133p53 was used to substitute OCT4. To generate LV-shΔ133p53, LV-shp53 and LV-shSTD, the promoter EF1a in LV-OCT4-Egfp plasmid was replaced with human U6 promoter. LV-OCT4-Egfp was digested with BamH1 and Nhe1, and OCT4-Egfp was replaced with different specific small hairpin DNA fragments synthesized by Invitrogen. The target sequence of *p53* shRNA is 5′-CAAUGGUUCACUGAAGACC-3′ from exon 4 and *Δ133p53* shRNA targets to 5′-CUUGUGCCCUGACUUUCAA-3′ from intron 4 were as described^21^. Sequences of different small hairpin DNA fragments were listed in Supplementary Table S1.

### RNA Analysis and qRT-PCR

For quantitative real-time reverse transcriptional PCR (qRT-PCR), total RNA was treated with DNaseI prior to reverse transcription and purified with RNeasy mini kit (QIAGEN). First strand cDNA was synthesized using M-MLV Reverse Transcriptase (Invitrogen). Reaction was performed in CFX96^TM^ Real-Time System (Bio-Rad) using SsoFast EvaGreen Supermix (Bio-Rad) according to the manufacturer’s instructions. Total RNA was normalized with *β-actin*. Statistics was obtained from three repeat experiments. Primers sequences used are listed in Supplemental Table S1.

### Immuno-blotting

For Western blotting, total protein was extracted using standard SDS sample buffer. Western blotting was performed as described^21^.

Rabbit monoclonal antibodies against human RAD51 (#5181-1), RAD52 (#5257-1), β-Actin (#1854-1) were from Epitomics. Rabbit polyclonal against human p53 CM1 (NCL-p53-CM1) was from Novocastra. Human P53 (DO-1) was from Santa Cruz Biotechnology. Mouse monoclonal antibody against human Lig4 (DR1085) was from Millipore. Goat polyclonal antibody against human OCT4 (sc-8628) and NANOG (AF1997) were from Santa Cruz Biotechnology and R & D.

### Immunofluorescence-staining

To analyze RAD51 and γH2AX foci formation in reprogamming cells, CCD-1079sk cells were reprogramed with Yamanaka 4 factors or combined with other factors as described in the section of Lentiviral transduction and reprogramming culture. At 9 and 12 dpi, cells were collected and washed with hES culture medium and then plated on a Coverglass For Growth (Fisher Scientific, FIS12-545-82) which were covered with gelatin. After 6 h of culture, cells were rinsed with PBS and incubated with Permeate Buffer (0.2% Triton X-100 in PBS) at room temperature for 3 min. Cells on coverslips were rinsed twice with ice-cold PBS and then fixed with 4% PFA (Sigma) on ice for 15 mins. Cells were washed twice with PBS, and permeabilized with PBST (0.2% triton X-100 in PBS) at room temperature (RT) for 15 mins. After blocking in FDB (0.2% Triton X-100, 2% donkey serum, 3% bovine serum albumin, 1 × PBS) for 30 mins at RT, the coverslips were incubated with primary antibody for 1 hour (h) at RT, followed by 3 × 3 mins washes with PBST. A secondary antibody (Invitrogen) (1:400 diluted in blocking solution) was added and incubated for a further 1 h at RT. After a total 3 rounds of washing with PBST quickly, the coverslips were mounted on slides with a mount medium containing DAPI (VectaShield). RAD51 polyclonal antibody (ct-1201, Cell Application) and γH2AX S139 monoclonal antibody (#05-636, Millpore) were used for immunostaining. Total number of γ-H2AX/Rad51 positive cells were counted from randomly picked up 150 cells in each sample.

iPS colony immunostaining was performed as described above. The antibodies were used as follow: Anti-SOX2 (rabbit IgG, 1:1000, Millipore, AB5603), Anti-SSEA4 (mouse IgG, 1:400, DSHB, MC-831-70), Anti-Nanog (goat IgG, 1:150, R&D, AF1997), Anti-Tra-1-60 (mouse IgM, 1:150, Millipore, MAB4360), Anti-Tra-1-81 (mouse IgM, 1:150, Millipore, MAB4381), Anti-mouse IgM (546 nm, goat IgG, 1:1000, Invitrogen, A21045), Anti-goat IgG (594 nm, donkey IgG, 1:1000, Invitrogen, A11058), Anti-mouse IgG (647 nm, donkey IgG, 1:1000, Invitrogen, A31571), Anti-rabbit IgG (647 nm, donkey IgG, 1:1000, Invitrogen, A31573).

### FACS

For FACS analysis in reprogramming cells, at 9 and 12 dpi, cells were sampled and stained with 7-AAD/Annexin V by Annexin V PE Apoptosis Detection Kit (eBioscience, 88-8102) according to manufacturer’s instruction. FACS analysis was carried out with a FACS Calibur Flow Cytometer (BD Biosciences). The raw data was statistically analyzed with Flowjo 7.6 and Microsoft Excel 2007.

### Lentivirus preparation

Dulbecco’s modified Eagle’s Medium (DMEM; Invitrogen) supplemented with 10% fetal bovine serum (FBS; Hyclone) was used to culture human 293 T cells in 75 cm^2^ flask covered with gelatin. 293 T cells at the density about 2 × 10^7^ to 3 × 10^7^/flask were transfected with 10 μg LV plasmids and 2 helper plasmids (7.5 μg pdR8.91 and 5 μg pVSVG) with 56 μl FuGENE HD (Roche) according to manufacturer’s instructions. After 24 hpt, culture medium was substituted with DMEM supplemented with 10% FBS. At 48 and 72 hpt, the culture medium containing viral particles was collected and filtrated with 0.22 μm filter. To measure virus titer, 1.5 × 10^5^ CCD-1079sk cells were infected with 1 μl viral particle and 0.1% polybrene in a 6-well plate. 2 days after, cells were stained with DAPI solution and photographed with fluorescence microscopy (Nikon Eclipse TE2000-S). The titer of each virus solution was calculated as the formula: Titer = 1.5 × 10^8^ × [Number of EGFP^+^ cells in a sight]/[Total number of nucleus in a sight] (virus/ml).

### Lentiviral transduction and reprogramming culture

CCD-1079sk cells at passage 6 were cultured in DMEM medium supplemented with 10% FBS. 2.0 × 10^5^ cells were transduced with a cocktail of lentivirus carrying 4 Yamanaka factors, or combined with a lentivirus shSTD, shp53, shΔ133p53 and shp53 plus Δ133p53 separately. Transduction medium were supplemented with 0.1% polybrene, and the day was defined as “0 Day post infection (dpi)”. The infected cells were plated to a 6-well plate. At 24 hour post infection (hpi), the medium was changed to fresh DMEM medium (with 10% FBS). At 2 dpi, cells were transfered to a new 6-well plate covered with mouse embryonic fibroblast (MEF) feeder cells and cultured for another 3 days. The medium was replaced with human stem cell medium (hES medium; Invitrogen) for each of 2-days. After 12 dpi, the medium was substituted with a mixed medium consisting of hES medium, Condition Medium (CM; Invitrogen) and basic fibroblast growth factor (bFGF; Invitrogen) (hES: CM = 1:1, bFGF 2 ng/ml) in each of 2-days. At 20 dpi, the formed iPS colonies were subjected to AP Staining. Around 25–30 dpi, the colonies were picked out for expansion growth with hES medium in a 48-well plate covered with MEF feeder cells. Finally, iPS colonies were cultured in a 25 cm^2^ flask with feeder cells for other experiments or storage. The cryopreservation media for iPS colonies consisted of 20% qualified embryonic stem cell FBS (GIBCO), 70% hES medium and 10% DMSO.

### AP staining

At day 20 dpi, reprogramming cell colonies was stained with Alkaline Phosphotase (AP) Staining Kit (Sidansai) as manufacturer’s instruction. AP positive colonies in each well were photographed with Sony W570 camera and the number of colonies in each well was counted for statistical analysis.

### Karyotype analysis

At 25–30 dpi, more than 5 reprogramming cell colonies from each treatment were separately picked into a new 12-well plate for further expansion. At passage 4, part of cells of each colony were subjected to AP staining and immunostaining with different iPS marker genes. Five AP and iPS marker positive colonies from each treatment were selected for continuing culture. About 2 × 10^7^ cells from each colony were sent to ADICON Clinical Lab INC (Hangzhou) for karyotype analysis. In each iPS clone, 25 metaphases and about 1000 chromosomes were observed. Average abnormal chromosome events came from 5 independent iPS clones in each treatment.

### Teratoma formation

IPS cells (four factors, or four factors plus Δ133p53) (10^6^ cells) were subcutaneously injected into irradiated (4 Gy) nude mice (injections were performed 1 day after irradiation). Teratomas were surgically removed or after 9 weeks of injection. Tissue was fixed in formalin at 4°C, embedded in paraffin wax, and sectioned at a thickness of 5 mm. Sections were stained with haematoxylin and eosin for pathological examination.

## Additional Information

**How to cite this article**: Gong, L. *et al*. p53 isoform Δ133p53 promotes efficiency of induced pluripotent stem cells and ensures genomic integrity during reprogramming. *Sci. Rep*. **6**, 37281; doi: 10.1038/srep37281 (2016).

**Publisher’s note:** Springer Nature remains neutral with regard to jurisdictional claims in published maps and institutional affiliations.

## Supplementary Material

Supplementary Information
