# Supplementary material for: p53 isoform Δ133p53 promotes efficiency of induced pluripotent stem cells and ensures genomic integrity during reprogramming
Source: Sci Rep. 2016 Nov 22;6:37281. doi: 10.1038/srep37281 (PMC5118801; doi:10.1038/srep37281)
Supplement: Supplementary Information [file srep37281-s1.pdf]

p53 isoform Δ133p53 promotes efficiency of induced pluripotent stem cells and ensures genomic integrity during reprogramming

Lu Gong[1,4†], Xiao Pan[1†], Haide Chen[2], Lingjun Rao[2], Yelin Zeng[2], Honghui Hang[3], Jinrong Peng[2*], Lei Xiao[2*], Jun Chen[1*]

[1] Key laboratory for Molecular Animal Nutrition, Ministry of Education, Innovation Center for Signaling Network, College of Life Sciences, Zhejiang University, Hangzhou 310058, China

[2]College of Animal Sciences, Zhejiang University, Hangzhou 310058, China

[3] Key Laboratory of Freshwater Fish Reproduction and Development, Ministry of Education, State Key Laboratory Breeding Base of Eco-Environments and Bio-Resources of the Three Gorges Reservoir Region, School of Life Sciences, Southwest University, 2 Tiansheng Road, Beibei, Chongqing, China 400715.

[4]Department of Genetics and Complex Diseases, Harvard School of Public Health, Boston, Massachusetts 02115, USA

Author Information: The authors declare no competing financial interest.

*Correspondence to: Jun Chen, Tel/Fax: 86-571-88982101, E-mail: chenjun2009@zju.edu.cn; Lei Xiao, Email: leixiao@zju.edu.cn; Jinrong Peng, Email: pengjr@zju.edu.cn.

[†]These two authors contributed equally

SUPPLEMENTARY INFORMATION

Supplementary Figures

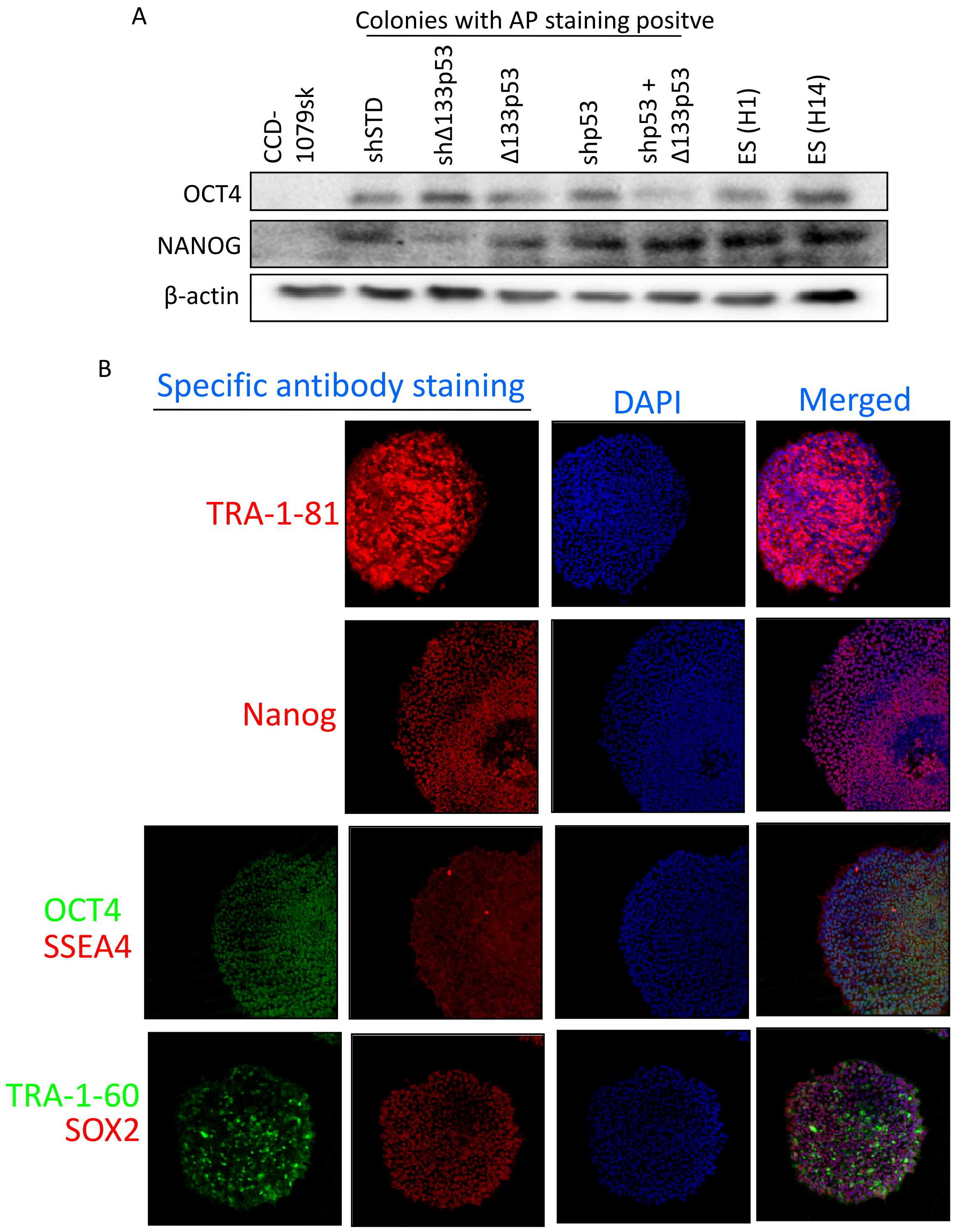
A
Colonies with AP staining positve
CCD-1079sk
shSTD
shΔ133p53
Δ133p53
shp53
shp53 + Δ133p53
ES (H1)
ES (H14)
OCT4
NANOG
β-actin
B
Specific antibody staining
DAPI
Merged
TRA-1-81
Nanog
OCT4
SSEA4
TRA-1-60
SOX2

Figure S1

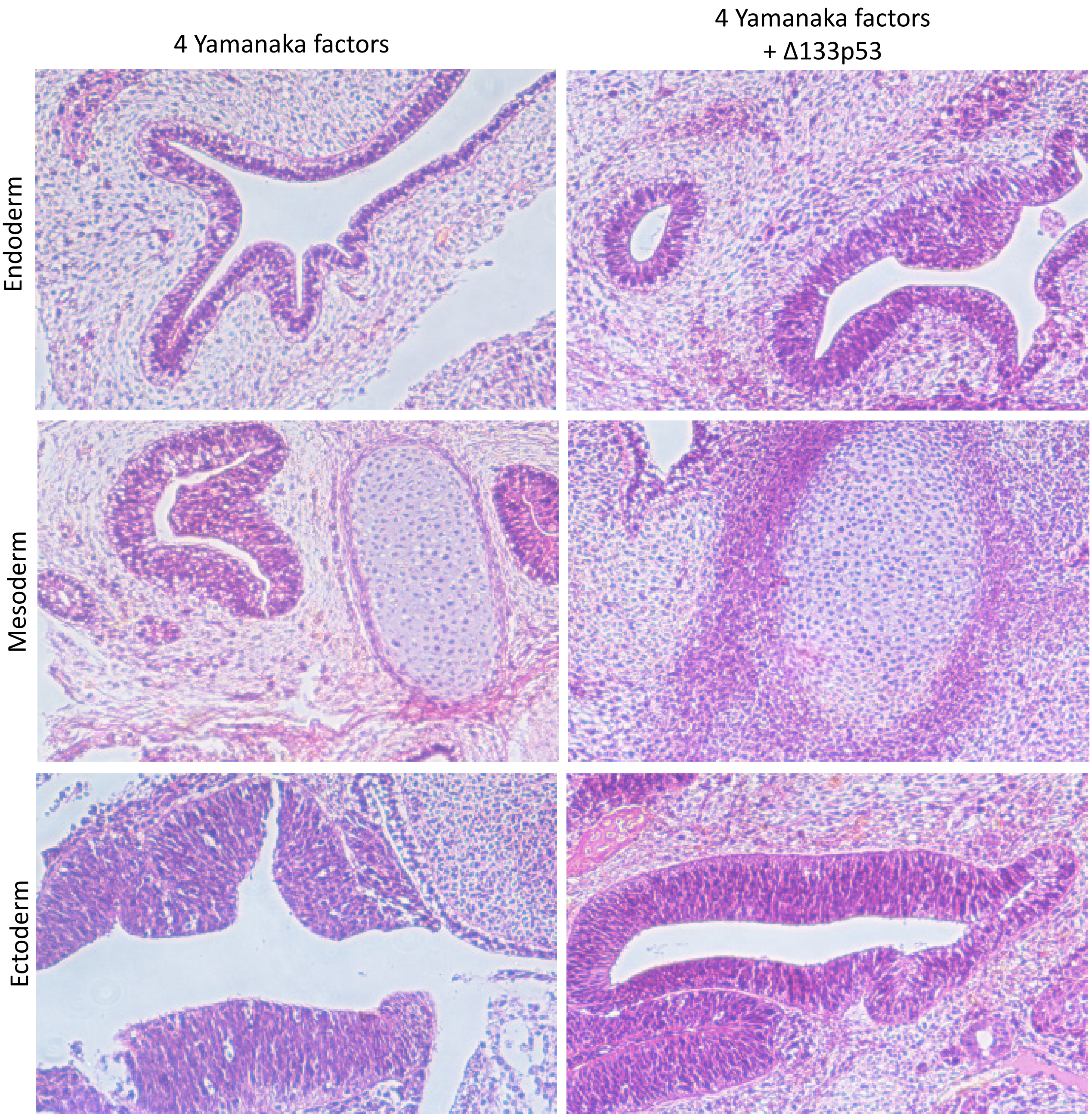
4 Yamanaka factors
4 Yamanaka factors
+ Δ133p53
Endoderm
Mesoderm
Ectoderm

Figure S2

SUPPLEMENTARY FIGURE LEGENDS

Supplementary Figure 1 Confirmation on characteristics of iPS cell colonies used in karyotype analysis. More than 5 reprogramming cell colonies from each treatment were separately picked into a new 12-well plate for further expansion at 25-30 dpi. At passage 4, part of cells were subjected to AP staining. AP positive colonies were used for further analysis. (a) Western blot analysis of iPS cell marker gens: OCT4 (upper panel) and NANOG (middle panel) in the AP positive colonies. CCD-1079sk cells were used as the negative control. Human ES cells of lines H1 and H14 were used as the positive control. (b) Immuno-staining or co-immuno-staining of iPS cell marker genes in the AP positive colonies as indicated. 1st panel: TRA-1-81 in red; 2nd panel: Nanog in red; 3rd panel: OCT4 in green and SSEA4 in red; 4th TRA-1-60 in green and SOX2 in red. DAPI for nuclear staining in blue.

Supplementary Figure 2 The injections of iPS cells induced by 4 Yamanaka factors or by 4 factors plus with Δ133p53 produced correspondingly 3 teratomas each. Histological analysis of haematoxylin and eosin stained sections revealed features of (from top to bottom): endoderm (respiratory epithelial-like tissue), mesoderm (cartilage-like tissue), and ectoderm (neuroepithelial-like tissue).

Supplementary Table S1: PCR Primers

**Primers for lentivirus constructs**	
Hu Δ 133p53-BamH1-For	CGCTGGATCCACCATGTTTTGCCAACTGGCCAAGA
Hu Δ 133p53-Nhe1-Rev	ACGTGCTAGCTCAGTCTGAGTCAGGCCCTTCTGT
Xho1-U6-For	CGTA CTCGAGGGTACCAAGGTCGGGCAGGA
U6-BamH1-sh Δ 133p53-Sal1- Rev	TAGCGTCGACAAAAACTTGTGCCCTGACTTTCAATCTC TTGAATTGAAAGTCAGGGCACAAGCCCGGATCCCGGT GTTTCGTCCTTTCCACA
Oligo-of-shp53-Cloning-For	GATCCGGGCAATGGTTCACTGAAGACCTTCAAGAGAG GTCTTCAGTGAACCATTGTTTTTG
Oligo-of-shp53-Cloning-Rev	TCGACAAAAACAATGGTTCACTGAAGACCTCTCTTGAA GGTCTTCAGTGAACCATTGCCCG
Oligo-of-shSTD-Cloning-For	GATCCGGGACGTGACACGTTCGGAGAATTCAAGAGATT

	CTCCGAACGTGTCACGTTTTTG
Oligo-of-shSTD-Cloning-Rev	TCGACAAAAAACGTGACACGTTCGGAGAATCTCTTGAATTCTCCGAACGTGTCACGTCCCG
**Primers for qRT-PCR in human cell line**	
Hu *d133p53*qPCR i4 For	TGGGTTGCAGGAGGTGCTTAC
Hu *d133p53*qPCR i4 Rev	CCACTCGGATAAGATGCTGAGG
Hu Full *p53*qPCR For	TGGAGGAGCCGCAGTCAGAT
Hu Full *p53*qPCR Rev	GCAGGGGCCGCCGGTGTAGGAG
Hu *β-Actin* qPCR For	TGGTGGGCATGGGTCAGAAGGAT
Hu *β-Actin* qPCR Rev	CCAGAGGCGTACAGGGATAGCAC
